# Syllable retrieval precedes sub-syllabic encoding in Cantonese spoken word production

**DOI:** 10.1371/journal.pone.0207617

**Published:** 2018-11-20

**Authors:** Andus Wing-Kuen Wong, Jie Wang, Siu-San Wong, Hsuan-Chih Chen

**Affiliations:** 1 Nam Shan Psychology Laboratory, Department of Social and Behavioural Sciences, City University of Hong Kong, Hong Kong S.A.R., China; 2 Department of Psychology, The Education University of Hong Kong, Hong Kong S.A.R., China; 3 Department of Psychology, The Chinese University of Hong Kong, Shatin, N.T., Hong Kong S.A.R., China; Leiden University, NETHERLANDS

## Abstract

Two experiments were conducted to investigate the time course of syllabic and sub-syllabic processing in Cantonese spoken word production by using the picture-word interference task. Cantonese-speaking participants were asked to name individually presented pictures aloud and ignore an auditory word distractor. The targets and distractors were either phonologically related (i.e., sharing two identical word-initial phonemes) or unrelated. In Experiment 1, the target syllables were all consonant-vowel (CV)-structured. The phonological distractor was either a CV syllable (i.e., Full Syllable Overlap) or a CVC (consonant-vowel-consonant) syllable (i.e., Sub-syllable Overlap). Relative to the unrelated control, Full Syllable Overlap distractors facilitated naming in all stimuli onset asynchronies (SOAs) (-175, 0, or +175 ms) whereas Sub-syllable Overlap distractors exhibited facilitation only at 0-ms and +175-ms SOAs. Experiment 2 adopted a similar design to examine the possible influence of syllabic structure similarity on the results of Experiment 1. The target syllables were all CVC-structured. The phonological distractor was either a CVC (i.e., Syllable-structure Consistent) or CV (i.e., Syllable-structure Inconsistent) syllable. Comparable priming was observed between the two distractor conditions across the three SOAs. These results indicated that an earlier priming effect was observed with full syllable overlap than sub-syllabic overlap when the degree of segmental overlap was held constant (Experiment 1). The earlier syllable priming observed in Experiment 1 could not be attributed to the effect of syllabic-structure (Experiment 2), thereby suggesting that the syllable unit is important in Cantonese and is retrieved earlier than sub-syllabic components during phonological encoding.

## Introduction

One contentious theoretical issue in the language production literature concerns the nature or size of the phonological unit being retrieved following lexical access, and whether this is language-universal or language-specific [1]. A widely accepted view is that, at least for Germanic languages such as Dutch and English, the first selectable phonological unit is a phoneme (or segment). Data from speech error studies showed that a majority of the phonological slips of the tongue involves a single segment [2], whereas errors involving only a single phonological feature (e.g., /təˈmeɪtoʊ/ -> / pəˈneɪtoʊ / for the word “tomato”, where the error involves an exchange of the place feature) are relatively rare [3]. Studies employing the form-preparation paradigm have provided convergent evidence [4]. Typically, participants were asked to produce repeatedly a small set of words that were either phonologically related or unrelated. Their naming responses were faster, relative to an unrelated condition, when the words shared the same word-initial segment but not when they shared only the same word-initial feature [5]. Similarly, by using a masked priming task, facilitation in naming latency has been reported when primes and targets shared an identical segment, and the size of priming was positively associated with the degree of segmental overlap, irrespective of the syllabic structure [6, 7]. Also, syllable units (as selectable chunks) have been found to have a functional role to play during the stage of phonetic encoding following phonological encoding [8]. Not surprisingly, most prominent models of speech production developed primarily upon Indo-European languages have unanimously emphasized the importance of phonemes during the transition from meaning to form [9].

However, a different picture was observed in non-Indo-European languages such as Chinese and Japanese. Using a similar form-preparation paradigm, researchers did not find a comparable effect of a single phoneme in Chinese or Japanese. Instead, significant priming effects were observed only when the words shared the same atonal syllable (i.e., syllable without the tone specified) in Mandarin [10, 11] or mora in Japanese [12]. Relatedly, Chen [13] observed a higher than chance level of speech errors involving the movement of the entire atonal syllable in Mandarin. Therefore, some researchers have proposed the proximate unit hypothesis, assuming that the nature of the first selectable phonological unit (i.e., proximate unit) varies across languages. Proximate units were proposed to be phonemes in Dutch and English, moras in Japanese, and atonal syllables in Mandarin [14].

Although the behavioral effect of a single segment has rarely been found in Chinese speech production [10, 15], there is evidence to suggest that sub-syllabic units are also implicated in Chinese phonological encoding. For instance, using the picture-word interference (PWI) task, Wong and Chen [16, 17] asked participants to name individually presented pictures aloud and ignore an accompanying word distractor. Significant facilitation effect on naming latency, relative to an unrelated control, was observed when the target and distractor shared the same sub-syllabic components, such as the syllable body, rhyme, or two identical phonemes. Similarly, other researchers have reported sub-syllabic priming effects in Chinese character naming among Mandarin-English bilinguals using a masked priming task [18].

To explain the robust effect of atonal syllables and the null effect of a single segment in Chinese speech production, the proximate unit hypothesis assumes that syllable retrieval precedes sub-syllabic encoding in Chinese. As phonemic specification is sub-ordinated to syllable retrieval, the sub-syllabic effects in Chinese word production are either weak or difficult to observe. This assumption has been included in the Chinese version of the Word-form Encoding and Activation Verification (WEAVER) model [19].

One key assumption of the above account is the order between syllabic and sub-syllabic processing in Chinese word production. However, evidence supporting this key assumption is scarce. In a recent attempt to investigate the time course of syllabic and sub-syllabic processing in overt Mandarin word production, Wang, Wong, & Chen [20] employed the PWI task and manipulated the stimuli onset asynchronies (SOAs). The distractor was visually presented either before (SOA = -100 ms), at (SOA = 0 ms), or after (SOA = +100 ms) the presentation of the target picture. Relative to an unrelated control, distractors sharing the same atonal syllable with the target facilitated naming latencies when SOAs = -100 or 0 ms. In contrast, significant priming was observed in the body-related condition only at 0-ms SOA. The relatively early syllabic and late sub-syllabic effects have been taken to support the notion that syllable retrieval precedes sub-syllabic encoding in Mandarin. It should be noted however, that in that study, the degree of segmental overlap between target and distractor was higher in the syllable-related condition (mean number of overlapping phonemes = 3.25) than that in the body-related condition (mean number of overlapping phonemes = 2.00). Given there is evidence to suggest that the size of priming observed in a production task is correlated with the degree of segmental overlap between prime and target [6] (but see also [21] and [22]), whether the relatively early effect of syllable priming was due to the unique role of the syllable or to the relatively higher degree of segmental overlap remains unclear.

The proximate unit hypothesis [14] is an attention-grabbing proposal in language production research as it could potentially account for many of the cross-language differences observed in the past [4, 5, 10, 12, 15]. However, there is yet no clear evidence to support an important assumption of the theory, that is, syllable selection occurs prior to sub-syllabic encoding in Chinese. Therefore, two Cantonese PWI experiments were conducted in which the degree of segmental overlap between target and distractor was kept constant across conditions. Three SOA conditions (-175, 0, and +175 ms) were adopted. Based on a comprehensive mega study on the previous speech production literature, Indefrey and Levelt [23] (see also [24]) estimated that the phonological encoding process starts at approximately 275 ms post target and lasts for around 180 ms before the phonetic encoding stage begins (assumed an average naming latency of 600 ms). Therefore, the chosen range of SOAs (with a total of 350 ms in duration) should be broad enough to cover the phonological encoding process (taking into account that the average naming latency in a PWI task is usually longer than 600 ms). Experiment 1 included a typical syllable-related distractor condition where the target (e.g., /maa5/ “horse”) and distractor (e.g., /maa4/) shared the same atonal syllable, which was consonant-vowel (CV)-structured. Distractors sharing the same CV component with the target but with an additional coda (e.g., /maan5/) were also included. Note that there are six possible codas in Cantonese and two in Mandarin. These two types of phonological distractor shared the same number of identical phonemes with their target (i.e., two identical phonemes), and the only difference between the two was whether the overlap constituted a full syllable overlap. According to the proximate unit hypothesis [14], an earlier facilitation effect should be observed in the full syllable overlap condition (i.e., CV distractors) than in the sub-syllable overlap condition (i.e., CVC distractors). Furthermore, as each written Chinese character maps directly onto a syllable unit in speech, to prevent potential bias to syllabic processing when deriving phonology from written Chinese, auditory distractors were used in this PWI study.

Nevertheless, one possible confound in Experiment 1 was that the two phonological conditions also differed in their syllabic structure. By using a masked priming-naming task, Verdonschot et al. [18] found significant onset priming effects in Mandarin speech production among highly proficient Mandarin-English bilinguals only when the prime and target had the same syllabic structure (e.g., /bi1/ and /ba1/) but not when the two had different syllabic structures (e.g., /bin1/ and /ba1/). This finding suggested that syllabic structure might have an influence on the phonological effects observed in a speech production task (see also [25]). Experiment 2 was therefore designed to examine whether similarity in syllabic structure between target and distractor would have an effect on the present PWI results.

## Experiment 1: Syllable vs. sub-syllabic component

This experiment aims at investigating the time course of syllabic and sub-syllabic processing in Cantonese phonological encoding using a PWI task while the degree of segmental overlap was manipulated across conditions.

### Materials and methods

#### Participants

Thirty Cantonese-speaking undergraduates (28 females; aged between 18 and 24 years old; mean age = 20.2±1.2 years) from the City University of Hong Kong (CityU) participated. They were all neurologically healthy, without a known history of speech or hearing impairment, and had normal or corrected-to-normal vision. The experiments reported here were approved by the Human Subjects Ethics Sub-Committee of the Research Committee of the City University of Hong Kong. Participants completed the experiment individually in a sound-attenuated room and each received an honorarium of HKD 50 (~USD 7).

#### Stimuli and apparatus

**Fig 1:**
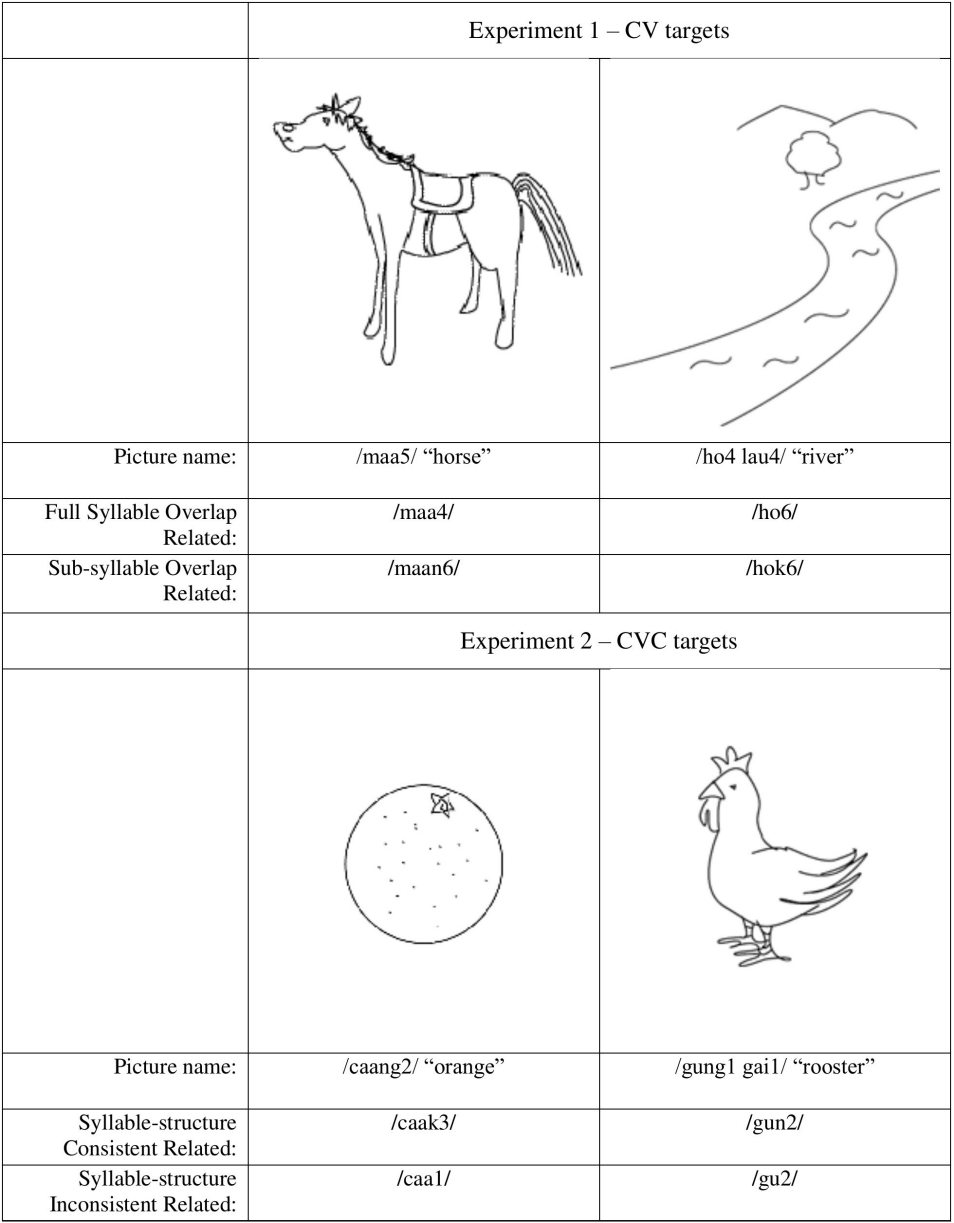
Samples of picture stimuli and distractors used in Experiments 1 and 2.

Thirty black-on-the-white line drawings of common objects adopted from previous related studies [16, 26] were used as picture stimuli. Twenty-one pictures had a disyllabic Cantonese name beginning with a CV syllable (e.g., /ho4 lau4/, meaning “river”). The remaining nine pictures had a mono-syllabic Cantonese name with a similar CV structure (e.g., /maa5/, meaning “horse”). More di-syllabic than mono-syllabic targets were included because the former is more common in modern Chinese [27]. Each picture was paired with two phonologically related mono-syllabic Cantonese word distractors (both had CV components similar to their target). For di-syllabic targets, the overlap between target and distractor always occurred in the word-initial syllable position. One distractor was a CV syllable (e.g., /ho6/ or /maa4/, which was consistent with the corresponding target /ho4 lau 4/ or /maa5/, respectively, in the Full Syllable Overlap condition) and the other one was a consonant-vowel-consonant (CVC) syllable (e.g., /hok6/ or /maan6/, which was inconsistent with the target /ho4 lau 4/ or /maa5/, respectively, in the Sub-syllable Overlap condition). Distractors were presented auditorily. The two sets of distractors were closely matched in syllable frequency and number of homophones [28], *t*s <1. Since Cantonese syllables with a stop coda (e.g., /p/, /t/ and /k/) are generally shorter in their acoustic duration relative to those without, the mean duration of the CVC distractors (468±116ms) was shorter than that of the CV distractors (547±54 ms), *t*(58) = 2.87, *p* < .05. Furthermore, an unrelated control condition was created for the Full Syllable Overlap condition by re-pairing the distractors with the targets within the same condition so that the distractor was unrelated to the target in each pair. A similar unrelated control condition was created for the Sub-syllable Overlap condition by re-pairing the distractors with the targets. Consequently, four distractor conditions were included, namely Full Syllable Overlap Related, Full Syllable Overlap Unrelated, Sub-syllable Overlap Related, and Sub-syllable Overlap Unrelated (see Supporting Information, S1 File, for details). The target and distractor in each pair were orthographically unrelated in their written form and were not semantically related in any obvious way. Samples of stimuli are shown in Fig 1.

The pictures were shown on a computer screen with a size of approximately 5° x 5° in visual angle. The auditory distractors were recorded by a female Cantonese native speaker, digitized with a sampling rate of 20000Hz, and presented to the participants through Sony MDR-NC 20 Noise Canceling headphones. Stimuli presentation was controlled by a computer running the DMDX program [29]. Participants’ vocal responses were recorded via a microphone connected to the same computer. The computer recorded the onset of each response to the nearest millisecond (ms) by a voice onset key.

#### Design and procedure

The experiment adopted a 3 (SOA: -175 ms, 0 ms, or +175 ms) x 2 (Distractor Type: Full Syllable Overlap vs. Sub-syllable Overlap) x 2 (Target-distractor Relatedness: Related vs. Unrelated) within-participants design. Each participant named each of the 30 pictures under each SOA and distractor condition, comprising a total of 360 (30 pictures x 3 SOAs x 4 distractor conditions) trials. Six blocks of 60 trials each were formed. Each picture appeared twice in each block. The four distractor conditions were intermixed and equiprobable in each block. Trial order within each block was pseudorandomized with no picture repetition in two consecutive trials. The block presentation order was randomized across participants. The experiment consisted of a learning and a testing phase. In the learning phase, participants were shown on the screen the 30 pictures individually along with their names. Afterward, the picture names were tested immediately to check if participants could name the pictures correctly. In the subsequent testing phase, each trial started with a centered fixation point presented for 1000 ms, followed by a 500-ms blank. The target picture was then presented and stayed on the screen until a naming response was detected or after a lapse of 5000 ms. Depending on the SOA condition, the auditory distractor was presented before (SOA = -175 ms), at (SOA = 0 ms), or after (SOA = +175 ms) the onset of the picture presentation. Participants’ naming latencies were measured from the time the picture was displayed. The next trial began after a 1000-ms blank. Participants were asked to name the target picture aloud as accurately and quickly as possible, and ignore the accompanying distractor. Prior to the start of the experiment, participants learned and named 10 pictures, which were not included in the experimental session, for practice. The entire experiment lasted 45 minutes.

### Results

Incorrect responses (0.8%), trials in which the voice key malfunctioned or mistriggered (1.6%), and outlier responses, which were 2.5 standard deviations beyond condition means (2.7%), were discarded from the reaction time analyses. Table 1 shows the participants’ mean naming latencies across conditions in Experiment 1.

**Table 1:** Participants’ mean naming latencies (M, in ms) across conditions in Experiments 1 and 2.

	SOA	Distractor Condition	M	SD	Difference
Expt. 1	- 175 ms	FSO-Re	710	117	26
		FSO-Un	736	116	
		SsO-Re	745	132	-11
		SsO-Un	734	115	
	0 ms	FSO-Re	738	122	41
		FSO-Un	779	129	
		SsO-Re	749	136	29
		SsO-Un	778	130	
	+175 ms	FSO-Re	757	142	37
		FSO-Un	794	145	
		SsO-Re	760	142	30
		SsO-Un	790	149	
Expt. 2	- 175 ms	SC-Re	655	98	27
		SC-Un	682	110	
		SI-Re	648	94	36
		SI-Un	684	106	
	0 ms	SC-Re	656	102	50
		SC-Un	706	113	
		SI-Re	663	108	42
		SI-Un	705	122	
	+175 ms	SC-Re	670	106	32
		SC-Un	702	116	
		SI-Re	674	106	28
		SI-Un	702	120	

Note: FSO-Re = Full Syllable Overlap Related; FSO-Un = Full Syllable Overlap Unrelated; SsO-Re = Sub-syllable Overlap Related; SsO-Un = Sub-syllable Overlap Unrelated; SC-Re = Syllable-structure Consistent Related; SC-Un = Syllable-structure Consistent Unrelated; SI-Re = Syllable-structure Inconsistent Related; SI-Un = Syllable-structure Inconsistent Unrelated. Difference = Unrelated—Related.

The remaining naming latency data were first inverse transformed (-1000/RT) and then submitted to linear mixed-effect modeling (LMEM) [30] implemented in R Version 3.4.3 [31]. We used lmerTest package [32] to conduct *F* tests on the fixed effects and calculate *p* values with Satterthwaite approximation to prevent Type 1 error (see [33], for details). SOA (-175, 0, or +175 ms), Distractor Type (full syllable or sub-syllable overlap), Target-distractor Relatedness (related or unrelated), and all possible interactions were fixed effects, while participants and items were random effects. Target Type (Mono-syllabic vs. Di-syllabic) and times of picture repetition were entered to control for possible confounds. Following the recommendations by Barr et al. [34], we tested the maximal model that converged and the final model included by-participant and by-item random intercepts, as well as by-participant random slopes for SOA, Distractor Type, and Target-distractor Relatedness: [invRT ~ SOA*typ*rel+syl+rep+ (1|item) + (1+SOA+typ+rel|participant)]. Results of *F* tests on the fixed effects using Satterthwaite approximation are shown in Table 2.

**Table 2:** Results of *F* tests on the fixed effects using Satterthwaite approximation in Experiments 1 and 2.

		Sum of Square	Mean Square	df numerator	df denominator	*F*	*p*	
Expt. 1	SOA	1.4	0.7	2	54.3	11.74	< .001	***
	typ	0.22	0.22	1	35.4	3.69	0.063	†
	rel	3.88	3.88	1	35.4	65.05	< .001	***
	SOA x typ	0.35	0.17	2	10125.8	2.89	0.056	
	SOA x rel	1.45	0.73	2	10125.6	12.14	< .001	***
	typ x rel	0.72	0.72	1	10125.8	12.09	< .001	***
	SOA x typ x rel	0.34	0.17	2	10125.8	2.83	0.059	†
Expt. 2	SOA	0.39	0.19	2	29.7	2.62	0.09	†
	typ	0.01	0.01	1	224.3	0.18	0.67	
	rel	5.61	5.61	1	29	75.45	< .001	***
	SOA x typ	0.04	0.02	2	10020.9	0.24	0.79	
	SOA x rel	0.7	0.35	2	10020.6	4.74	0.009	**
	typ x rel	0.02	0.02	1	10020.1	0.2	0.65	
	SOA x typ x rel	0.46	0.23	2	10020	3.08	0.046	*

*Note*. SOA (-175, 0, +175 ms); typ: Distractor Type (Full Syllable Overlap vs. Sub-syllable Overlap in Expt 1; Syllable-structure Consistent vs. Syllable-structure Inconsistent in Expt 2); rel: Target-distractor Relatedness (Related vs. Unrelated); syl: target type (Mono-syllabic vs. Di-syllabic); rep: times of repetition.

†0.05 < *p* < 0.1,

* *p* < 0.05,

** *p* < 0.01,

*** *p* < 0.001.

Similar to Wang et al. [20], the simple main effects of Target-distractor Relatedness were also analyzed separately for each SOA and Distractor Type condition: [invRT ~ rel+syl+rep+ (1|item) + (1+rel|participant)]. The unrelated control condition was treated as the baseline in simple effect analyses. The regression coefficients (*b*), standard errors (*SE*), and *p* values are reported in Table 3. Full Syllable Overlap distractors significantly facilitated naming responses across all SOAs, while Sub-syllable Overlap distractors showed significant facilitation at 0- and +175-ms SOAs only.

**Table 3:** Simple main effects of Target-distractor relatedness in Experiments 1 and 2.

	SOA	typ	*b*	*SE*	*t*	*p*	
Expt. 1	-175 ms	Full Syllable Overlap	-0.054	0.011	-4.79	< 0.001	***
		Sub-syllable Overlap	0.011	0.012	0.94	0.355	
	0 ms	Full Syllable Overlap	-0.084	0.013	-6.62	< 0.001	***
		Sub-syllable Overlap	-0.069	0.014	-5	< 0.001	***
	+175 ms	Full Syllable Overlap	-0.072	0.014	-5.1	< 0.001	***
		Sub-syllable Overlap	-0.053	0.013	-4.2	< 0.001	***
Expt. 2	-175 ms	Syllable-structure Consistent	-0.053	0.012	-4.33	< 0.001	***
		Syllable-structure Inconsistent	-0.085	0.013	-6.48	< 0.001	***
	0 ms	Syllable-structure Consistent	-0.12	0.017	-6.89	< 0.001	***
		Syllable-structure Inconsistent	-0.09	0.018	-5.15	< 0.001	***
	+175 ms	Syllable-structure Consistent	-0.079	0.016	-4.9	< 0.001	***
		Syllable-structure Inconsistent	-0.057	0.014	-3.91	< 0.001	***

### Discussion

Experiment 1 replicated the findings of previous Chinese PWI studies that a significant facilitation effect can be observed when the target and distractor shared the same atonal syllable or syllable body [16, 17, 20]. Notably, the present results showed that when the number of overlapping phonemes between target and distractor was held constant, an earlier priming effect was observed in the full syllable overlapping condition than the sub-syllable overlapping condition.

Furthermore, additional analyses were conducted (see Supporting Information, S1 File) to examine whether the distractors’ acoustic duration would have an effect on the phonological effects observed. The above LMEM analyses were repeated on a subset of 15 pictures with a relatively longer CVC distractor (with a mean duration of 554±69 ms, which was comparable to the duration of the CV distractors). Significant priming effects were found in the Sub-syllable Overlap condition only at the 0-ms and +175-ms SOAs but not when SOA = -175 ms, indicating that the observed time course difference between Full Syllable Overlap and Sub-syllabic Overlap conditions was not affected by the duration of the distractors.

## Experiment 2: Syllable-structure consistent vs. Syllable-structure inconsistent

In Experiment 1, the Full Syllable Overlap, but not the Sub-syllable Overlap, distractors matched with the targets in syllabic structure. Therefore, whether the observed time course difference can be attributed to the effect of similarity in syllabic structure remains unclear. Experiment 2 was conducted to assess this alternative account.

### Materials and methods

#### Participants

A separate group of 30 participants (29 females; aged between 19 and 30 years old; mean age = 21.9±2.7 years) from the same pool of participants in Experiment 1 was recruited for Experiment 2, and were paid (HKD 50) for their participation.

#### Stimuli and apparatus

Experiment 2 used the same apparatus, design, and procedure, as that of Experiment 1 but a different set of stimuli. Thirty black-on-the-white line drawings of common objects adopted from previous related studies [16, 26] were used in Experiment 2. Twenty pictures had a disyllabic Cantonese name beginning with a CVC syllable (e.g., /gung1 gai1/, meaning “rooster”). The remaining ten pictures had a mono-syllabic Cantonese name with a similar CVC structure (e.g., /caang2/, meaning “orange”). Each picture was paired with two phonologically related mono-syllabic Cantonese word distractors overlapping with the target the same word-initial CV components. For di-syllabic targets, the overlap between target and distractor always occurred in the word-initial syllable position. One distractor was a CVC syllable (e.g., /gun2/ and /caak3/, which were consistent with their corresponding targets /gung1 gai1/ and /caang2/; i.e., Syllable-structure Consistent) and the other was a CV syllable (e.g., /gu2/ or /caa1/, which were syllabically inconsistent with their targets /gung1 gai1/ and /caang2/; i.e., Syllable-structure Inconsistent). Distractors were presented auditorily and closely matched in syllable frequency and number of homophones across conditions. Similar to Experiment 1, the mean duration of the CVC distractors (375±121ms) was shorter than that of the CV distractors (516±71 ms), *t*(58) = 5.46, *p* < .05. Two corresponding unrelated control conditions were created by re-pairing the related distractors with the targets in the same condition. Consequently, four distractor conditions were included, namely Syllable-structure Consistent Related, Syllable-structure Consistent Unrelated, Syllable-structure Inconsistent Related, and Syllable-structure Inconsistent Unrelated. The target and distractor in each pair were also unrelated orthographically in their written form and were semantically unrelated (see Fig 1).

### Results

Incorrect responses (0.6%), trials in which the voice key malfunctioned or mistriggered (2.8%), and outlier responses that were 2.5 standard deviations beyond condition means (2.4%) were discarded from reaction time analyses. Table 1 shows the participants’ mean naming latencies across conditions in Experiment 2.

The same set of LMEM analyses were conducted on the inverse-transformed naming latencies. As Table 2 shows, the SOA x Distractor Type x Target-distractor Relatedness interaction was significant. Table 3 displays the simple main effects of Target-distractor Relatedness for each condition. Both types of related distractors significantly facilitated naming responses relative to the unrelated controls across all SOAs.

### Discussion

Although the two phonological conditions in Experiment 2 differed in their syllabic structure (one was consistent with the target and the other was not), both conditions exhibited similar patterns of phonological priming, suggesting that the effect of syllabic structure was minimal (if any) under the present PWI paradigm.

## General discussion

Two PWI experiments were conducted to investigate the time course of syllabic and sub-syllabic processing in Cantonese spoken word production. Importantly, the degree of segmental overlap between target and distractor was kept constant across phonological conditions. Facilitation effects were observed in Experiment 1 when the target and the distractor shared the same atonal syllable (i.e., Full Syllable Overlap) across all SOA conditions (-175, 0, and +175 ms). In contrast, a comparable facilitation effect was observed only in late SOAs (0 and +175 ms) when the target and the distractor shared the same word-initial phonemes but had different atonal syllables (i.e., Sub-syllable Overlap). These results indicated the unique role of the atonal syllable, which is retrieved earlier than its sub-syllabic components in Cantonese speech production. Furthermore, two types of phonological distractor with different syllabic structures were included in Experiment 2 where they both shared the same two word-initial phonemes with the CVC-structured targets. An identical pattern of priming was observed across the two phonological conditions, indicating that the time course difference observed in Experiment 1 was not because of the influence of the syllabic structure.

According to the proximate unit hypothesis, the first selectable phonological unit in Mandarin Chinese is atonal syllable [14, 19]. Sub-syllabic specification has been proposed to be subsequent to syllable retrieval. During the atonal syllable retrieval stage, only distractors that share the same atonal syllable should be able to affect this stage, this is because the size of the phonological unit concerned is syllable-based. In the subsequent sub-syllabic specification stage, distractors sharing a significant number of identical phonemes with the target should be able to induce a priming effect, irrespective of whether the target and the distractor also share the same entire syllable. This is because the phonological unit involved in this latter stage is phoneme-based. The results from Experiment 1 are largely consistent with these predictions. An early priming effect at -175-ms SOA was observed only in the full syllable overlap condition but not in the sub-syllable overlap condition. Comparable priming effects were also observed across the two phonological conditions in subsequent SOAs (0 and -175 ms). The former early effect was dependent on syllable overlap (syllable retrieval stage), whereas the latter comparable effects across conditions were dependent on the degree of segmental overlap (sub-syllabic encoding stage). Furthermore, the results of Experiment 2 indicated that the effect of syllabic structure (e.g., CV or CVC) is minimal, at least under the present PWI paradigm. This result was also consistent with the WEAVER model [35] which assumes that syllabic structures are not specified in the metrical frame (i.e., a representation for supra-segmental information).

Note, however, that significant effects of sub-syllabic overlap were observed in Experiment 2 when SOA = -175 ms, but not in Experiment 1 at the same -175-ms SOA. A similar situation has been reported and discussed in the past PWI literature. For instance, Schriefers, Meyer, and Levelt [36] investigated the time course of semantic and phonological processing in spoken word production using the PWI task with three SOA conditions including -150, 0, and +150 ms. A significant interference effect was observed with semantically related distractors at the -150-ms SOA, whereas a significant facilitation effect was observed with word-begin phonologically related distractors only at 0- and +150-ms SOAs. In another similar PWI study using the same three SOA conditions, Meyer and Schriefers [37] compared the time course of word-begin related phonological priming and word-end related phonological priming. Notably, significant word-begin related phonological facilitation could be observed at the -150-ms SOA. However, as discussed in Meyer and Schriefers [37], the *absolute* timing of the effects might be affected by many factors including participants’ overall naming speed, materials used, and experimental design; however, the more crucial and informative point to the theoretical questions might be the *relative* timing of the effects. While further research is needed to examine the absolute timing of the sub-syllabic effects, the relative timing of the phonological effects observed in Experiment 1 is consistent with the notion that syllabic processing begins earlier than sub-syllabic processing in Cantonese word production, and that such a pattern cannot be accounted for by the effect of syllabic structure priming (Experiment 2).

The current results and the relevant evidence available in the literature [10, 14, 20] are in general consistent with the view of Roelofs [19] that atonal syllable has its unique representation in Chinese speech production and is retrieved first prior to its sub-syllabic constituents. In addition, similar to Roelofs [19], the units being processed after atonal syllables have been selected are segments. This view is consistent with the present and the past studies showing sub-syllabic priming effects in Chinese speech production when the prime and target shared similar syllable-body [15, 17, 18, 20, 25, 38], rhyme [16, 17], or onset together with the same coda [38]. Although a significant behavioral onset priming effect has rarely been observed in Chinese before (but see [18], for an exception), there is electrophysiological evidence showing that the onset consonant is implicated in Chinese spoken word planning [39, 40]. Together, the available evidence points to two conclusions, 1) both atonal syllables and segments are legitimate processing units in Chinese speech production, and 2) atonal syllables play a more salient role than segments and are retrieved first prior to segmental specification in Chinese. And these two conclusions are consistent with the view of Roelofs [19]. To account for the null (behavioral) effect of onset in Chinese (which stands in marked contrast to many Indo-European languages such as Dutch and English where a robust effect of onset has repeatedly been found), Roelofs [19] made one further assumption in that segments are selected in parallel in Chinese following atonal syllable selection. Since segments are selected in parallel, priming the onset consonant alone would not result in any observable facilitation to the whole segmental specification process, because the system still needs to wait until all other segments have been processed. This hypothesis might be one possibility to account for the null effect of onset in the previous Chinese studies [10, 15, 16]. However, this hypothesis (i.e., parallel encoding of segments in Chinese) has not been vigorously investigated and there is yet no clear empirical evidence speaking to this issue. In fact, Wang et al. [20] has made an initial attempt to address this issue by comparing a body related condition (e.g., /da/ and /dao/) and a rhyme related condition (e.g., /ao/ and /dao/) in a PWI study with three different SOAs. And the results seemed to suggest that segments are encoded serially even in Mandarin Chinese, a similar conclusion of serial encoding of segments has been reached by previous studies on Indo-European languages (and hence Wang et al. argued that serial encoding of segments might be a language-universal feature). Indeed, more research is needed to investigate whether segments are encoded in parallel or serial in Chinese.

Nevertheless, a very important question remains, which is about what happens to the segments following atonal syllable selection that precludes the effect of onset but allows some form of sub-syllabic priming (e.g., syllable body or rhyme priming) in Chinese. We think this issue is still open. What we can see from the literature is that a single segment (e.g., onset consonant, nucleus, or coda) has rarely shown a significant priming effect in Chinese. Yet, significant priming effects have been replicated a number of times when multiple segments (not necessarily constituting to a syllable unit) are primed simultaneously [16–18, 20, 25, 38]. It has been suggested that participants’ L2 (English) proficiency might play a role and contribute to the observed sub-syllabic priming effects [18]. Regarding the possible influence of L2 on L1 speech production (see also [22], the present data do not speak clearly to this issue. This hypothesis might be possible. However, the mechanism of how a phoneme-based L2 (e.g., English) would affect the phonological encoding process of a syllable-based L1 (e.g., Chinese) is still largely unclear. Even if there are influences from L2 to L1 speech production, the same question remains, it is still not clear why significant effects are present when multiple segments are primed together but absent when only a single segment is primed in Chinese. There is limited evidence for this issue. One possibility might be that different types of phonological units have different “weightings” in determining the speed of phonological encoding. It might be the case that the proximate units (e.g., phonemes in Dutch and English, and atonal syllables in Chinese) bear a more significant weighting than other non-proximate phonological units (e.g., phonemes in Chinese). Priming of the proximate units would lead to (behaviorally) observable effects because the phonological encoding process is largely dependent on the readiness of the proximate units. Priming of a non-proximate unit, such as a single phoneme in Chinese, might also facilitate the phonological encoding process to some extent but the effect tend to be small or non-detectable (at least behaviorally). When multiple non-proximate units are primed together, the joint effect could be strong enough to reach the threshold and become detectable. Nevertheless, future research is warranted to examine this hypothesis.

Furthermore, to explain the null effect of a single phoneme in the past Chinese speech production studies, it has been proposed that Chinese speakers either could not or did not orient their attention to the non-proximate segmental units and hence could not benefit from segmental priming [14]. The present results indicate that even when the Chinese speakers can benefit from sub-syllabic (i.e., syllable-body) priming, a primary role of the syllable unit is still evident. The effect of the atonal syllable has been found to be larger [15: Experiment 3] or earlier (as the present results showed), than that of the sub-syllabic units, indicating the primacy role of the atonal syllable in spoken Chinese.

Previous research on language production has shown contrastive patterns with salient effects of a single phoneme frequently observed in Dutch or English but not in Mandarin or Cantonese [4, 5, 10, 15]. While robust effects of the syllable units have been reported in the latter, the functional role of the syllable has been found in the former only at the late phonetic encoding stage [8]. The proximate unit hypothesis is one unified account that can potentially explain these cross-language differences [14]. One key assumption of this theory concerns the time course of syllabic and sub-syllabic processing in Chinese phonological encoding. This issue, however, has not been unambiguously settled in the past as the degree of segmental priming was almost always varied between syllabic and sub-syllabic priming conditions. The present results offer new and clear evidence that syllable retrieval precedes sub-syllabic encoding in Cantonese, which is consistent with the theoretical claim that the first selectable phonological unit is language-specific.

## Supporting information

S1 File Item lists and additional analyses. (DOCX)

S2 File Data. (XLSX)
