# Supplementary material for: Syllable retrieval precedes sub-syllabic encoding in Cantonese spoken word production
Source: PLoS One. 2018 Nov 20;13(11):e0207617. doi: 10.1371/journal.pone.0207617 (PMC6245687; doi:10.1371/journal.pone.0207617)
Supplement: S1 File — (DOCX) [file pone.0207617.s001.docx]

**Supporting Information for *PLoS One***

Item lists and additional analyses

Table S1. Stimuli used in Experiment 1

| Picture name | |  |  | FSO-Re | Duration (in ms) |  | SSO-Re | Duration (in ms) |  | FSO-Un |  | SSO-Un |
| --- | --- | --- | --- | --- | --- | --- | --- | --- | --- | --- | --- | --- |
| 梳 | *comb* | /so1/ |  | /so4/ | 608 |  | /song3/ | 699 |  | /wu6/ |  | /caan2/ |
| 鼠 | *mouse* | /syu2/ |  | /syu4/ | 626 |  | /syun3/ | 641 |  | /haa2/ |  | /zik6/ |
| 士兵 | *soldier* | /si6 bing1/ |  | /si4/ | 568 |  | /sin1/ | 634 |  | /fo2/ |  | /haak3/ |
| 火 | *fire* | /fo2/ |  | /fo1/ | 594 |  | /fong1/ | 610 |  | /ci1/ |  | /wut6/ |
| 貨車 | *truck* | /fo3 ce1/ |  | /fo2/ | 561 |  | /fong2/ | 595 |  | /syu4/ |  | /gaan1/ |
| 叉 | *fork* | /caa1/ |  | /caa4/ | 452 |  | /caan2/ | 576 |  | /zi6/ |  | /syut3/ |
| 獅子 | *lion* | /si1 zi2/ |  | /si5/ | 610 |  | /sing4/ | 541 |  | /bo3/ |  | /baak6/ |
| 廚師 | *chef* | /cyu4 si1/ |  | /cyu5/ | 603 |  | /cyun1/ | 533 |  | /baa6/ |  | /song3/ |
| 馬 | *horse* | /maa5/ |  | /maa4/ | 503 |  | /maan6/ | 533 |  | /fo1/ |  | /gung1/ |
| 齒輪 | *gear* | /ci2 leon4/ |  | /ci1/ | 594 |  | /cin4/ | 522 |  | /syu1/ |  | /fong1/ |
| 咖啡 | *coffee* | /gaa3 fe1/ |  | /gaa1/ | 494 |  | /gaan1/ | 498 |  | /cyu5/ |  | /cyun1/ |
| 耳 | *ear* | /ji5/ |  | /ji4/ | 506 |  | /jin4/ | 498 |  | /ngaa3/ |  | /syun3/ |
| 匙羹 | *spoon* | /ci4 gang1/ |  | /ci3/ | 566 |  | /cim1/ | 483 |  | /wu3/ |  | /bong2/ |
| 蘑菇 | *mushroom* | /mo4 gu1/ |  | /mo2/ | 545 |  | /mong5/ | 475 |  | /jyu2/ |  | /faat3/ |
| 河流 | *river* | /ho4 lau4/ |  | /ho6/ | 571 |  | /hok6/ | 473 |  | /si5/ |  | /fuk6/ |
| 牙 | *tooth* | /ngaa4/ |  | /ngaa3/ | 441 |  | /ngaam1/ | 468 |  | /gu3/ |  | /wun2/ |
| 紙巾 | *napkin* | /zi2 gan1/ |  | /zi6/ | 543 |  | /zing3/ | 467 |  | /caa4/ |  | /ngaam1/ |
| 鎖鑰 | *key* | /so2 si4/ |  | /so1/ | 598 |  | /sok3/ | 464 |  | /fu4/ |  | /cin4/ |
| 樹木 | *tree* | /syu6 muk6/ |  | /syu1/ | 591 |  | /syut3/ | 460 |  | /ci3/ |  | /cim1/ |
| 花朵 | *flower* | /faa1 do2/ |  | /faa3/ | 601 |  | /faat3/ | 448 |  | /so4/ |  | /sing4/ |
| 鼓 | *drum* | /gu2/ |  | /gu3/ | 550 |  | /gung1/ | 448 |  | /maa4/ |  | /maan6/ |
| 菠蘿 | *pineapple* | /bo1 lo4/ |  | /bo3/ | 536 |  | /bong2/ | 425 |  | /zi3/ |  | /zing3/ |
| 烏龜 | *turtle* | /wu1 gwai1/ |  | /wu3/ | 580 |  | /wun2/ | 417 |  | /ji4/ |  | /fong2/ |
| 乳豬 | *suckling pig* | /jyu5 zhu1/ |  | /jyu2/ | 457 |  | /jyun2/ | 406 |  | /ho6/ |  | /sok3/ |
| 蝦 | *shrimp* | /haa1/ |  | /haa2/ | 545 |  | /haak3/ | 355 |  | /mo2/ |  | /jin4/ |
| 斧頭 | *axe* | /fu2 tau2/ |  | /fu4/ | 559 |  | /fuk6/ | 348 |  | /si4/ |  | /jik1/ |
| 二胡 | *Erhu* | /ji6 wu2/ |  | /ji5/ | 459 |  | /jik1/ | 301 |  | /so1/ |  | /mong5/ |
| 蝴蝶 | *butterfly* | /wu4 dip2/ |  | /wu6/ | 591 |  | /wut6/ | 270 |  | /gaa1/ |  | /sin1/ |
| 芝士 | *cheese* | /zi1 si2/ |  | /zi3/ | 520 |  | /zik6/ | 236 |  | /faa3/ |  | /hok6/ |
| 巴士 | *bus* | /baa1 si2/ |  | /baa6/ | 445 |  | /baak6/ | 232 |  | /ji5/ |  | /jyun2/ |

Note: Picture names are presented in traditional Chinese characters. Their English translations are presented in italics. The number besides each syllable marking denotes the tone of that syllable. FSO-Re = Full-Syllable Overlap Related condition; FSO-Un = Full-Syllable Overlap Unrelated control; SSO-Re = Sub-Syllable Overlap Related condition; SSO-Un = Sub-Syllable Overlap Unrelated control.

Table S2. Stimuli used in Experiment 2

| Picture name | |  |  | SC-Re | Duration (in ms) |  | SI-Re | Duration (in ms) |  | SC-Un |  | SI-Un |
| --- | --- | --- | --- | --- | --- | --- | --- | --- | --- | --- | --- | --- |
| 公雞 | *rooster* | /gung1 gai1/ |  | /gun2/ | 427 |  | /gu2/ | 437 |  | /wok6/ |  | /ci5/ |
| 蛋糕 | *cake* | /daan6 gou1/ |  | /daam1/ | 440 |  | /daa1/ | 376 |  | /jim4/ |  | /si2/ |
| 工人 | *worker* | /gung1 jan4/ |  | /guk6/ | 163 |  | /gu3/ | 490 |  | /cit3/ |  | /si3/ |
| 漢堡 | *hamburger* | /hon3 bou2/ |  | /hok6/ | 407 |  | /ho6/ | 536 |  | /wut6/ |  | /syu6/ |
| 青蛙 | *frog* | /cing1 waa1/ |  | /cit3/ | 318 |  | /ci5/ | 551 |  | /fok3/ |  | /ngaa4/ |
| 風扇 | *fan* | /fung1 sin3/ |  | /fuk6/ | 316 |  | /fu4/ | 635 |  | /gon2/ |  | /waa4/ |
| 潛艇 | *submarine* | /cim4 teng5/ |  | /cin2/ | 500 |  | /ci1/ | 475 |  | /jyut6/ |  | /baa2/ |
| 樂譜 | *music score* | /ngok6 pou2/ |  | /ngong5/ | 518 |  | /ngo1/ | 443 |  | /daam1/ | | /ci1/ |
| 城堡 | *castle* | /sing4 bou2/ |  | /sit6/ | 409 |  | /si2/ | 571 |  | /baat3/ |  | /caa1/ |
| 荒島 | *desert island* | /fong1 dou2/ |  | /fok3/ | 394 |  | /fo3/ | 600 |  | /zek3/ |  | /ze6/ |
| 糖果 | *candy* | /tong4 gwo2/ |  | /tok3/ | 306 |  | /to5/ | 507 |  | /fuk6/ |  | /laa1/ |
| 滑鼠 | *mouse* | /waat6 syu2/ |  | /waan4/ | 370 |  | /waa4/ | 484 |  | /sing3/ |  | /ngo1/ |
| 箭靶 | *arrow target* | /zin3 baa2/ |  | /zik1/ | 186 |  | /zi3/ | 498 |  | /hok6/ |  | /ho6/ |
| 鉛筆 | *pencil* | /jyun4 bat1/ |  | /jyut6/ | 243 |  | /jyu5/ | 478 |  | /ngaat3/ | | /wo6/ |
| 皇冠 | *crown* | /wong4 gun1/ |  | /wok6/ | 318 |  | /wo6/ | 603 |  | /gun2/ |  | /daa1/ |
| 鋼琴 | *piano* | /gong3 kam4/ |  | /gon2/ | 476 |  | /go1/ | 455 |  | /cin2/ |  | /saa2/ |
| 纜車 | *cable car* | /laam6 ce1/ |  | /laan4/ | 500 |  | /laa1/ | 545 |  | /jing1/ |  | /ji2/ |
| 蠟燭 | *candle* | /laap6 zuk1/ |  | /laam4/ | 463 |  | /laa3/ | 615 |  | /syun2/ |  | /to5/ |
| 斑馬 | *zebra* | /baan1 maa5/ |  | /baat3/ | 261 |  | /baa2/ | 449 |  | /guk6/ |  | /fo3/ |
| 雪人 | *snowman* | /syut3 jan4/ |  | /syun2/ | 562 |  | /syu6/ | 653 |  | /ngong5/ | | /ji6/ |
| 橙 | *orange* | /caang2/ |  | /caak3/ | 311 |  | /caa1/ | 495 |  | /tok3/ |  | /zi3/ |
| 井 | *well* | /zeng2/ |  | /zek3/ | 251 |  | /ze6/ | 522 |  | /laam4/ |  | /wu6/ |
| 碗 | *bowl* | /wun2/ |  | /wut6/ | 217 |  | /wu6/ | 571 |  | /saam1/ | | /mo4/ |
| 鑽 | *drill* | /zyun3/ |  | /zyut6/ | 282 |  | /zyu2/ | 458 |  | /sit6/ |  | /go1/ |
| 傘 | *umbrella* | /saan3/ |  | /saam1/ | 533 |  | /saa2/ | 583 |  | /zik1/ |  | /zyu2/ |
| 葉 | *leaf* | /jip6/ |  | /jing1/ | 510 |  | /ji2/ | 420 |  | /laan4/ |  | /fu4/ |
| 眼 | *eye* | /ngaan5/ |  | /ngaat3/ | 212 |  | /ngaa4/ | 405 |  | /mok6/ |  | /gu2/ |
| 網 | *net* | /mong5/ |  | /mok6/ | 313 |  | /mo4/ | 519 |  | /zyut6/ |  | /gu3/ |
| 煙 | *cigarette* | /jin1/ |  | /jim4/ | 435 |  | /ji6/ | 513 |  | /caak3/ |  | /laa3/ |
| 骰 | *dice* | /sik1/ |  | /sing3/ | 621 |  | /si3/ | 609 |  | /waan4/ | | /jyu5/ |

Note: Picture names are presented in traditional Chinese characters. Their English translations are presented in italics. The number besides each syllable marking denotes the tone of that syllable. SC-Re = Syllable-structure Consistent Related condition; SC-Un = Syllable-structure Consistent Unrelated control; SI-Re = Syllable-structure Inconsistent Related condition; SI-Un = Syllable-structure Inconsistent Unrelated control.

To examine whether the null effect of Sub-syllable Overlap distractors at the -175-ms SOA condition in Experiment 1 was due to reason that the Sub-syllable Overlap (CVC) distractors were generally shorter in duration, relative to the Full Syllable Overlap (CV) distractors, similar LMEM analyses were conducted on a subset of the data from Experiment 1. This subset of data was derived from the 15 pictures (half of all picture stimuli used in the main experiment) that had a Sub-syllable Overlap (CVC) distractor with a relatively longer duration. The mean duration of the 15 Sub-syllable Overlap distractors involved was 554±69 ms, which was comparable to the mean duration of the CV distractors in the main experiment, 547±54 ms. Similar to Experiment 1, the model being tested included by-participant and by-item random intercepts, as well as by-participant random slopes for SOA, Distractor Type, and Target-distractor Relatedness: [invRT ~ SOA*typ*rel+syl+rep+ (1|item) + (1+SOA+typ+rel|participant)]. Results of *F* tests on the fixed effects using Satterthwaite approximation are shown in Table S3.

Table S3. Results of *F* tests on the fixed effects using Satterthwaite approximation based on a subset of data from Experiment 1

|  |  | Sum of Square | Mean Square | df numerator | df denominator | *F* | *p* |  |
| --- | --- | --- | --- | --- | --- | --- | --- | --- |
| Expt. 1 | SOA | 0.966 | 0.48 | 2 | 47.4 | 7.91 | 0.001 | ** |
| (subset) | typ | 0.024 | 0.02 | 1 | 39.2 | 0.4 | 0.53 |  |
|  | rel | 2.492 | 2.492 | 1 | 37.4 | 40.81 | < .001 | *** |
|  | SOA x typ | 0.402 | 0.201 | 2 | 5000.3 | 3.29 | 0.037 | * |
|  | SOA x rel | 0.967 | 0.484 | 2 | 4999.2 | 7.92 | < .001 | *** |
|  | typ x rel | 0.394 | 0.394 | 1 | 4999.7 | 6.46 | 0.011 | * |
|  | SOA x typ x rel | 0.219 | 0.109 | 2 | 5000.2 | 1.79 | 0.167 |  |

*Note*. SOA (-175, 0, +175 ms); typ: Distractor Type (Full Syllable Overlap vs. Sub-syllable Overlap); rel: Target-distractor Relatedness (Related vs. Unrelated).* *p* < 0.05, ** *p* < 0.01, *** *p* < 0.001.

To further understand the pattern of full syllable and sub-syllable overlap priming based on this subset of data from Experiment 1, the simple main effects of target-distractor relatedness were further analyzed at each SOA and distractor type condition. The unrelated condition was treated as baseline in simple effect analyses. The results are shown in Table S4.

Table S4. Simple main effects of target-distractor relatedness based on a subset of data from Experiment 1

|  | SOA | typ | *b* | *SE* | *t* | *p* |  |
| --- | --- | --- | --- | --- | --- | --- | --- |
| Expt. 1 | -175 ms | Full Syllable Overlap | -0.059 | 0.02 | -3.26 | 0.0014 | ** |
| (subset) |  | Sub-syllable Overlap | 0.01 | 0.016 | 0.63 | 0.53 |  |
|  | 0 ms | Full Syllable Overlap | -0.094 | 0.017 | -5.52 | < 0.001 | *** |
|  |  | Sub-syllable Overlap | -0.088 | 0.021 | -4.26 | < 0.001 | *** |
|  | +175 ms | Full Syllable Overlap | -0.078 | 0.019 | -4.14 | < 0.001 | *** |
|  |  | Sub-syllable Overlap | -0.048 | 0.019 | -2.52 | 0.018 | * |

In addition, by using the full set of data from Experiment 1, additional analyses were performed to compare the two unrelated control conditions (i.e., Full Syllable Overlap Unrelated vs. Sub-syllable Overlap Unrelated; with different distractor durations) at each SOA condition [invRT ~ typ+syl+rep+ (1|item) + (1+typ|participant)]. No significant results were obtained in Experiment 1 (-175-ms SOA: *b* = 0.006, *SE* = 0.011, *t* = 0.51, *p* = 0.61; 0-ms SOA: *b* = 0.002, *SE* = 0.01, *t* = 0.22, *p* = 0.83; +175-ms SOA: *b* = 0.009, *SE* = 0.012, *t* = 0.7, *p* = 0.49). A similar analysis was performed on the data from Experiment 2 and no significant results were found (-175-ms SOA: *b* = -0.01, *SE* = 0.012, *t* = -0.78, *p* = 0.43; 0-ms SOA: *b* = 0.014, *SE* = 0.014, *t* = 1.06, *p* = 0.29; +175-ms SOA: *b* = 0.009, *SE* = 0.014, *t* = 0.67, *p* = 0.5). These results indicated that the duration of the distractors per se had minimal effect on the present PWI results.
